# Supplementary material for: Critical ionic transport across an oxygen-vacancy ordering transition
Source: Nat Commun. 2022 Sep 1;13:5130. doi: 10.1038/s41467-022-32826-8 (PMC9437025; doi:10.1038/s41467-022-32826-8)
Supplement: Supplementary file 1 — Supplementary Information [file 41467_2022_32826_MOESM1_ESM.pdf]

Supplementary Information

Critical ionic transport across an oxygen-vacancy ordering transition

J. S. Lim *et al.*

Supplementary Figures

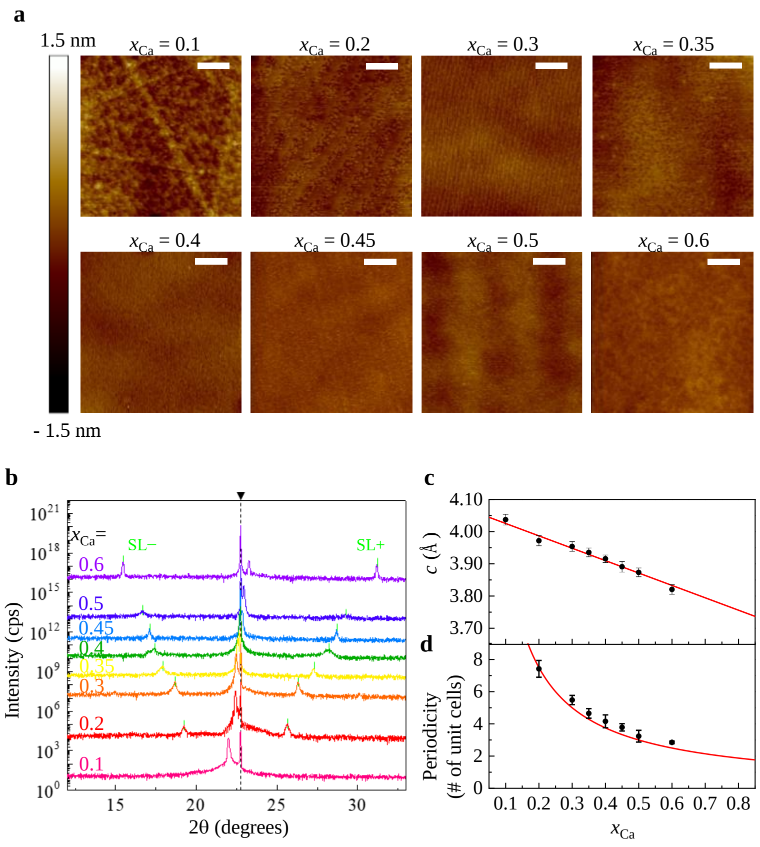

Supplementary Fig. 1: Structures of as-grown BCFO films on $SrTiO_3$ substrates. a, The surface topographic images of 100-nm-thick as-grown BCFO films on $SrTiO_3$ substrates have atomically flat surfaces. The surface roughnesses were evaluated to be less than 0.3 nm. The film of x_{Ca} = 0.1 has a monoclinic structure with ferroelectricity similar to $BiFeO_3$. Other BCFO films at x_{Ca} = 0.2 or higher have pseudo-tetragonal structures. Scale bars represent 1 μm. **b**, X-ray 2θ-ω scans at room T for samples with varying x_{Ca} (0.1 ~ 0.6). Dashed line represents the (001) peak of $SrTiO_3$. BCFO films at x_{Ca} = 0.2 or above have superlattice peaks (green lines, SL±), indicating the periodic appearance of V_O layers along the c axis. **c**, c-axis lattice parameter versus x_{Ca}. The red line denotes the linear interpolation between the lattice parameters of $BiFeO_3$ and $CaFeO_{2.5}$ according to Vegard's law. Error bars were estimated to be 1/5 of the FWHMs of the BCFO film peaks. **d**, Periodicity of ordered V_O layers versus x_{Ca}. Error bars were estimated from the FWHMs of the SL± peaks. Red line denotes an empirical rule, i.e., $n_{period} = 1.5/x_{Ca}$. As the distance between V_O layers becomes closer, it tends to deviate from the empirical rule. BCFO films of x_{Ca} = 0.2 (0.3) have n_{period} = 7 or 8 (5 or 6) unit cells, but for x_{Ca} = 0.6, most of the V_O layers are ordered with a periodicity of 3 unit cells.

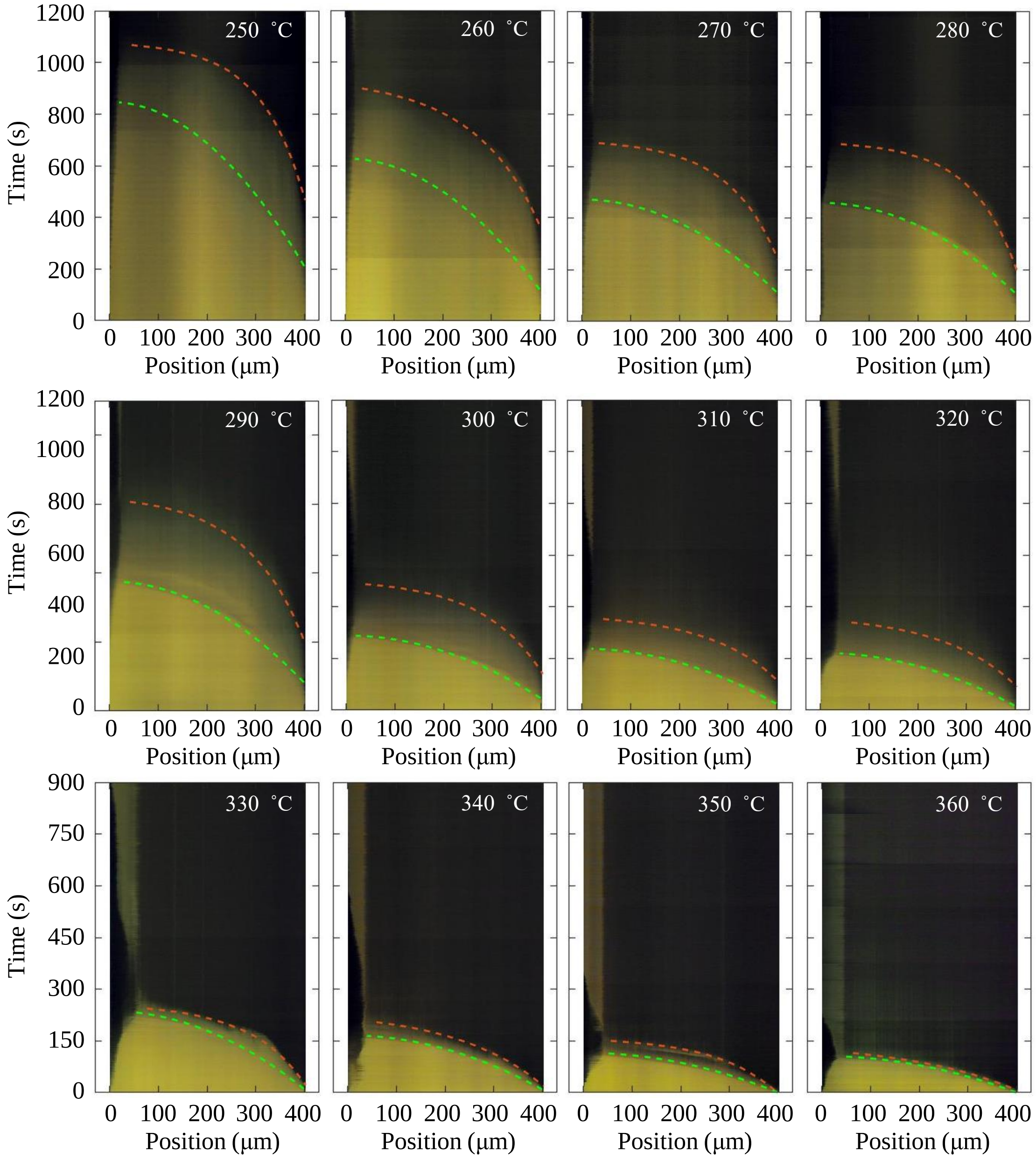

Supplementary Fig. 2: *T* dependence of the electroforming process for x_{Ca} = 0.45. We applied an external bias of 25 V to the BCFO channel. The real-time evolution of optical color contrast along the centerline of the BCFO channel was observed as a function of elapsed time at elevated *T*s from 250 °C to 360 °C at intervals of 10 °C. At high *T*s, V_Os were quickly migrated to establish the electrically-formed state. The color boundaries (dashed orange or green lines) between the oxygen-rich, intermediate, and oxygen-poor regions give a trajectory $z(t)$ of collective V_O migration, respectively. A large amount of V_Os were accumulated near the left electrode at position zero, showing a narrow and dark *n*-type region. The analysis of the green lines is summarized in Supplementary Information Section 8.

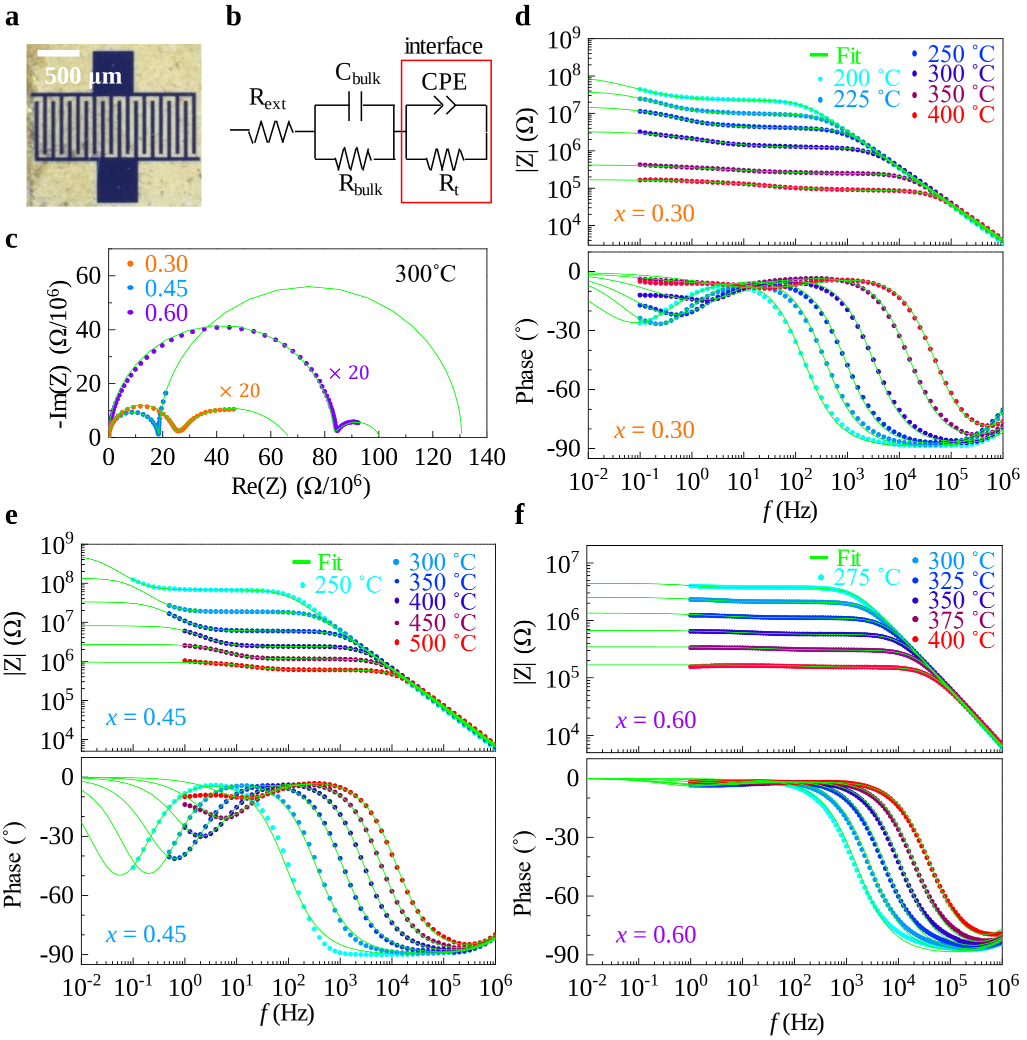

Supplementary Fig. 3: Impedance spectra of BCFO films. **a**, Microscopic image of platinum interdigitated electrodes on a BCFO film. **b**, The equivalent circuit of a BCFO channel between the interdigitated electrodes. **c**, Nyquist plots of the impedance spectroscopy data. **d**, **e** and **f**, Impedance spectroscopy data of BCFO films at x_{Ca} = 0.3, 0.45, and 0.6. The green lines represent the fitting curves to the equivalent circuit in b. Two semicircles are observed in each of the Nyquist plots in c. The first semicircle originates from the RC circuit (R_{bulk} and C_{bulk}) that describes the mixed conducting (electronic and ionic) property of a BCFO film. The second semicircle is due to the interfaces between the electrodes and BCFO film. To reflect the inhomogeneous ion distribution or slow adsorption process at the interfaces, both constant phase element (CPE) and charge-transfer resistance (R_t) are introduced to agree with the empirical result. In the mixed-conductor part, R_{bulk} can be expressed as $R_eR_{ion}/(R_e+R_{ion})$, where $R_{e(ion)}$ is electronic (ionic) resistance. In the low-frequency regime, the total resistance (R_{bulk} + R_t) can be interpreted as R_e due to ion-blocking electrodes.

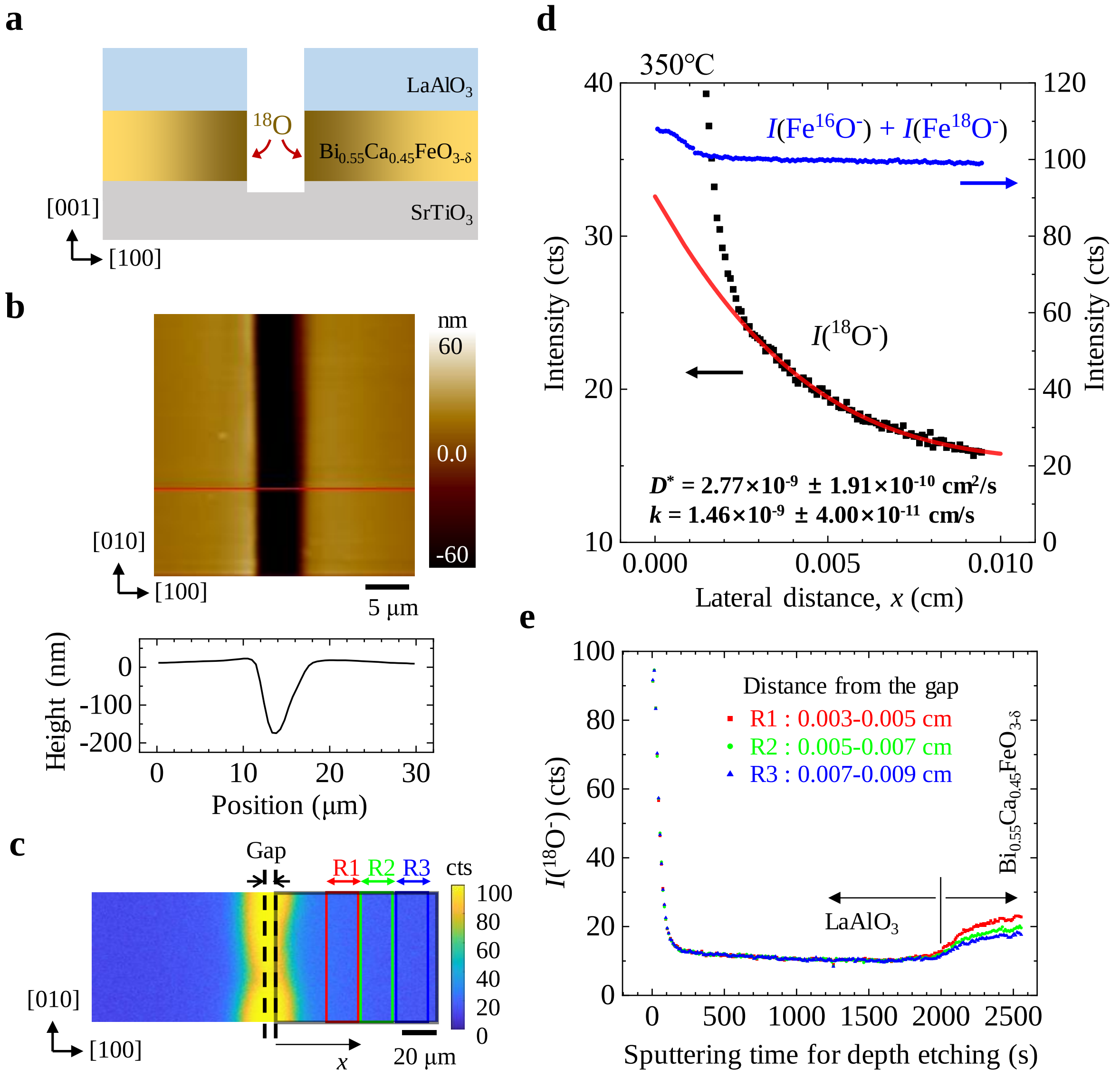

Supplementary Fig. 4: Oxygen isotope exchange profile analysis obtained by time-of-flight secondary ion mass spectroscopy (TOF-SIMS) for a BCFO film at x_{Ca} = 0.45. a, Schematic of the sample and lateral isotope exchange process through the gap (elongated along y-axis) created by mechanical scratching. The scratched 55-nm-thick BCFO thin film with a 50-nm-thick $LaAlO_3$ capping layer was annealed at 350 °C in an oxygen isotope (^{18}O) environment (at 500 Torr) for 90 minutes. **b**, Topographic image near the gap and the height profile across the gap (along the red line). **c**, Area (xy) mapping of $^{18}O^-$ intensity measured by TOF-SIMS after reaching the BCFO layer by in-situ depth etching of the capping layer. **d**, Line profiles (black points) of $^{18}O^-$ intensity along [100] over the region marked by the black rectangle in **c**. Blue points represent the sum of $Fe^{16}O^-$ and $Fe^{18}O^-$ intensities. A red curve was obtained by performing fitting over an area more than 0.003 cm away from the side of the gap. **e**, Depth (z-axis) profiles of $^{18}O^-$ intensity in each region (R1, R2 and R3) with different distances from the gap.

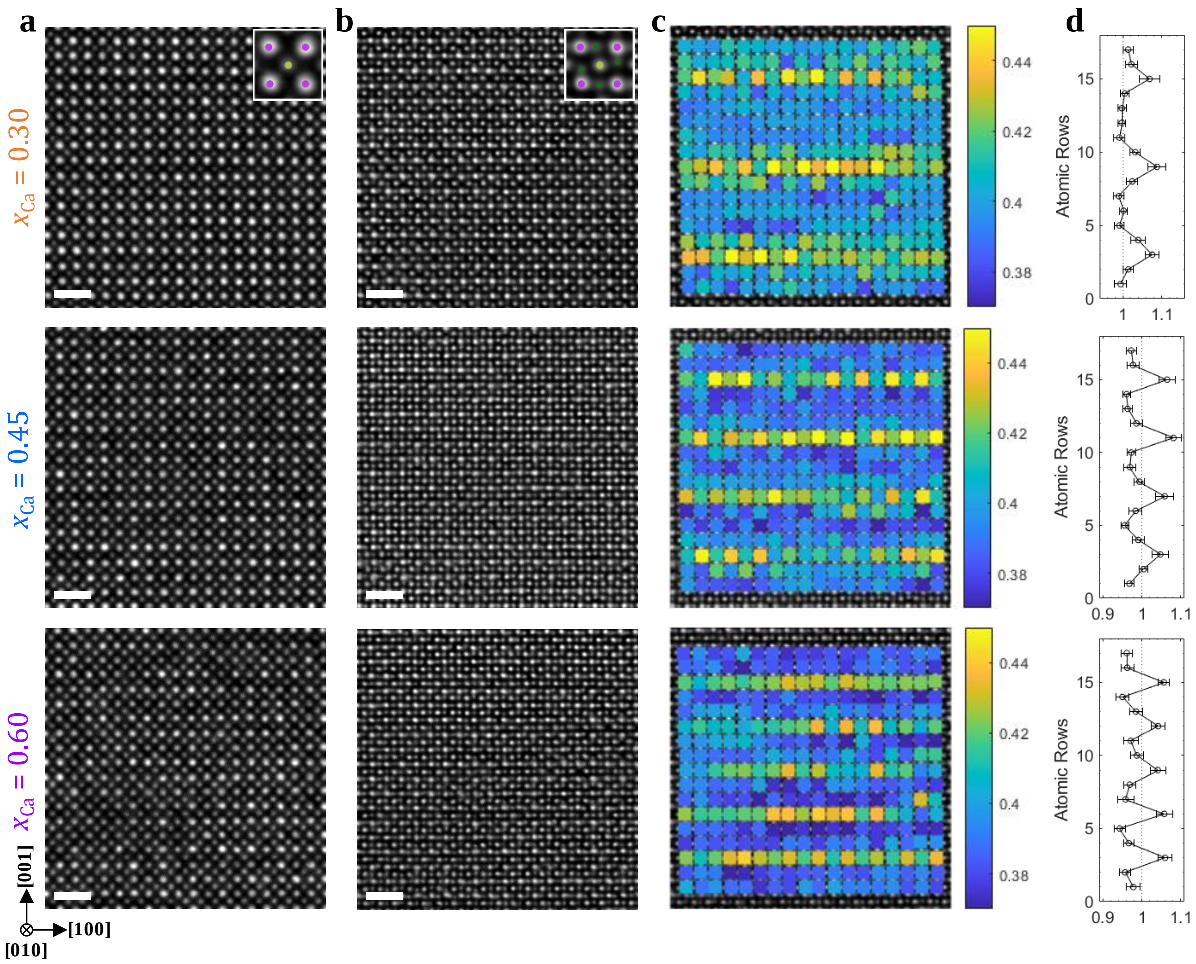

Supplementary Fig. 5: Observation of ordered V_O layers in BCFO films. a, Simultaneously acquired HAADF-STEM and **b**, inverted ABF-STEM images of the BCFO (x_{Ca} = 0.30, 0.45 and 0.60) films along the [010] zone axis. The magnified portions given in the insets show the pseudocubic structure with overlaid purple, yellow and green dots indicating the positions of the Bi/Ca, Fe/O and O atomic columns, respectively. **c**, Maps of the out-of-plane interatomic distances for the Bi/Ca sublattice as obtained by peak pair analysis. The V_O layers display a larger out-of-plane lattice parameter. The periodicity n_{period} is equal to ~6, 4 and 3 for the BCFO films with x_{Ca} = 0.30, 0.45 and 0.60, respectively. **d**, Profiles along [001] of the tetragonal c/a distortion. Scale bars represent 1 nm.

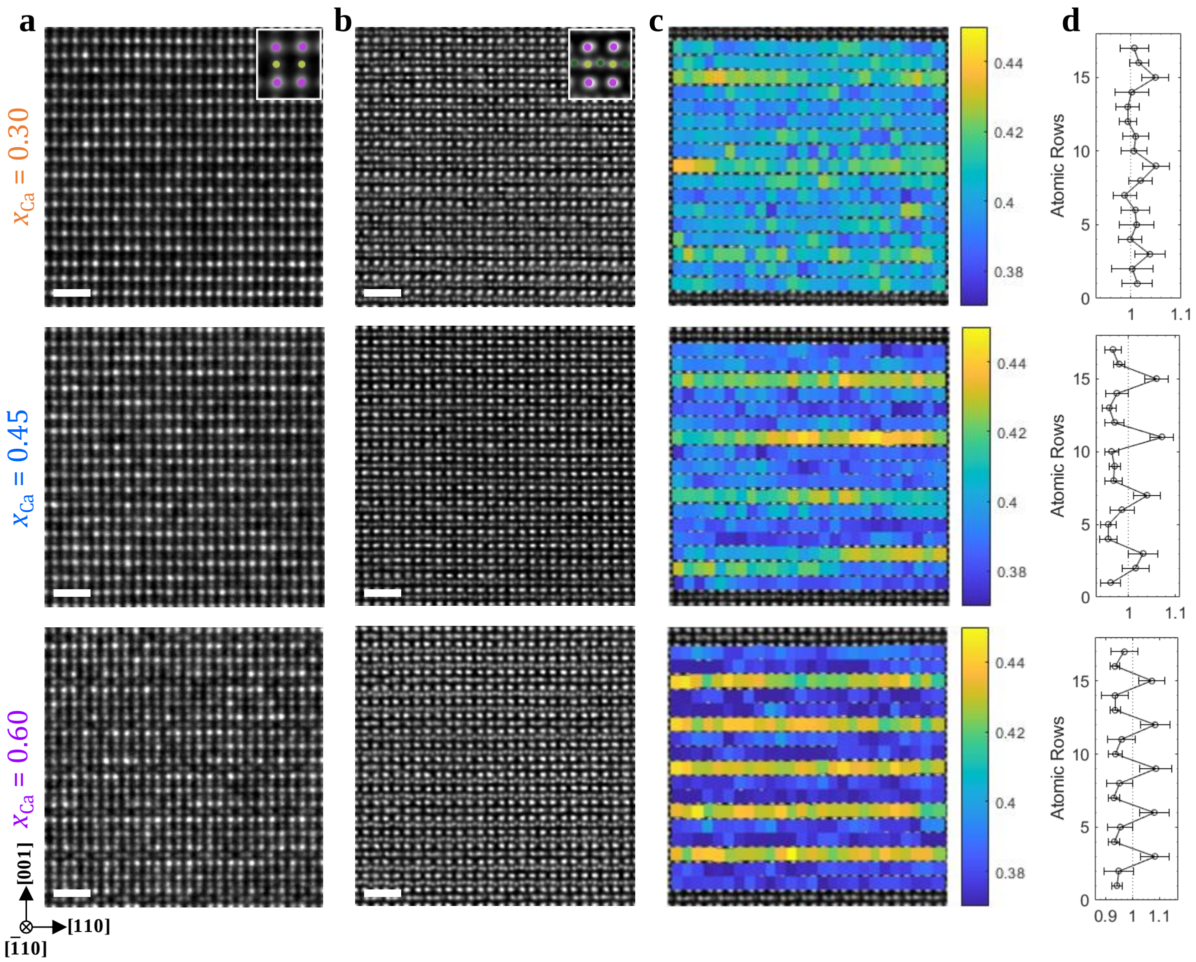

Supplementary Fig. 6: Observation of ordered V_O layers in BCFO films. **a**, Simultaneously acquired HAADF-STEM and **b**, inverted ABF-STEM images of the BCFO (x_{Ca} = 0.30, 0.45 and 0.60) films along the [$\bar{1}$10] zone axis. The magnified portions given in the insets show the pseudocubic structure with overlaid purple, yellow and green dots indicating the positions of the Bi/Ca, Fe and O atomic columns, respectively. **c**, Maps of the out-of-plane interatomic distances for the Bi/Ca sublattice as obtained by peak pair analysis. The V_O layers display a larger out-of-plane lattice parameter. The periodicity n_{period} is equal to ~6, 4 and 3 for the BCFO films with x_{Ca} = 0.30, 0.45 and 0.60, respectively. **d**, Profiles along [001] of the tetragonal c/a distortion. Scale bars represent 1 nm.

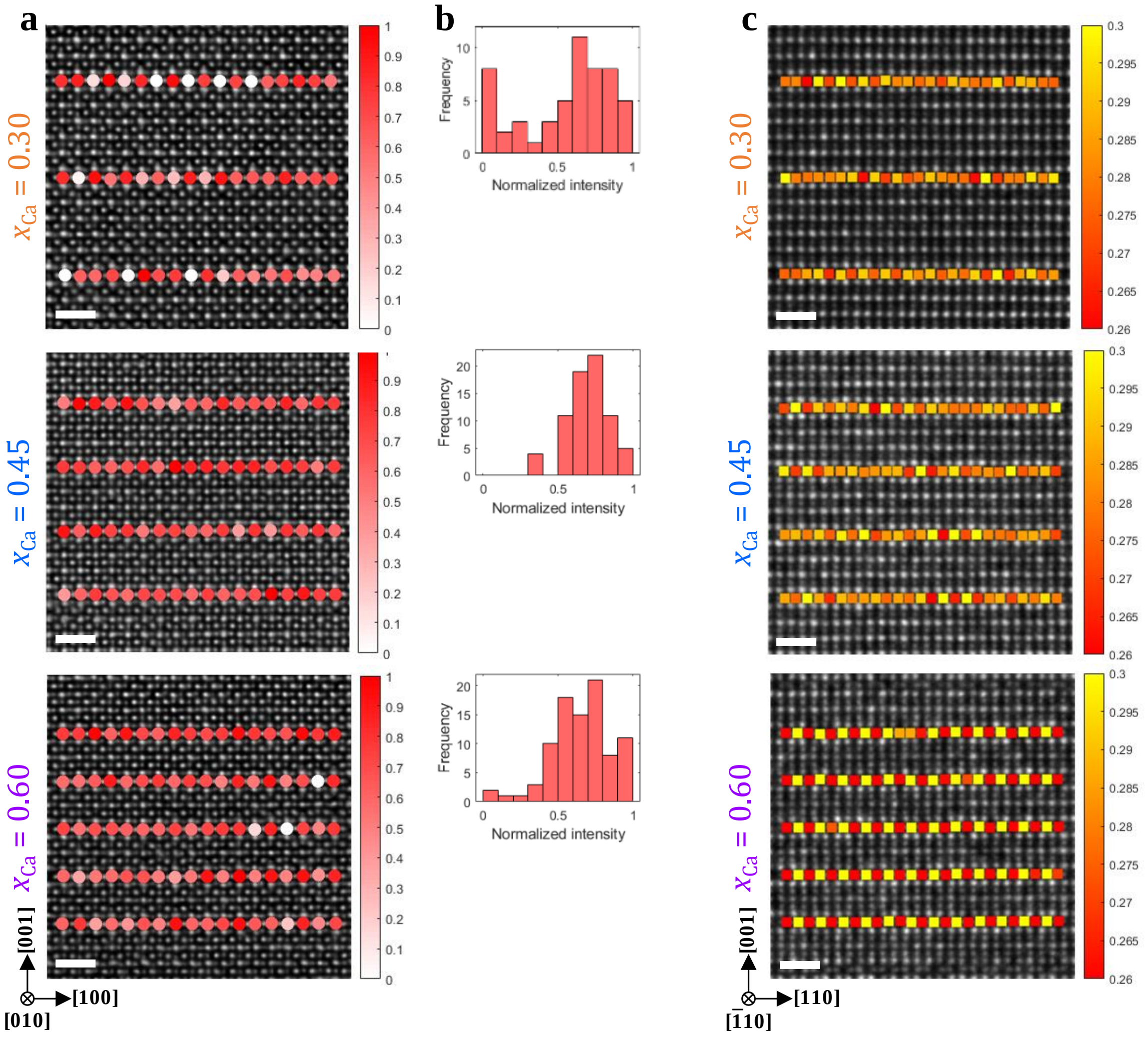

Supplementary Fig. 7: Observation of ordered V_O channels in BCFO films. a, Inverted ABF-STEM images of the BCFO (x_{Ca} = 0.30, 0.45 and 0.60) films along the [010] zone axis with extracted fit at the V_O layers of the experimental intensities for the O atomic columns plotted at their fitted coordinates. For comparison purposes, the fitted intensities of each film are normalized to the maximum atomic column intensity. **b**, Corresponding intensity histograms. For the x_{Ca} = 0.30 phase histogram, the presence of a second maxima centered at zero intensity reveals the occurrence of V_O channels. **c**, HAADF-STEM images of the BCFO films along the [$\bar{1}$10] zone axis with extracted fit at the V_O layers of the experimental Fe-Fe distances. Two clearly different Fe-Fe distances alternate along the [110] direction. The Fe-Fe distances are 0.27±0.01 and 0.29±0.01 nm for the x_{Ca} = 0.30 and 0.45 phases, and 0.24±0.02 and 0.33±0.02 nm for the x_{Ca} = 0.60 phase. Scale bars represent 1 nm.

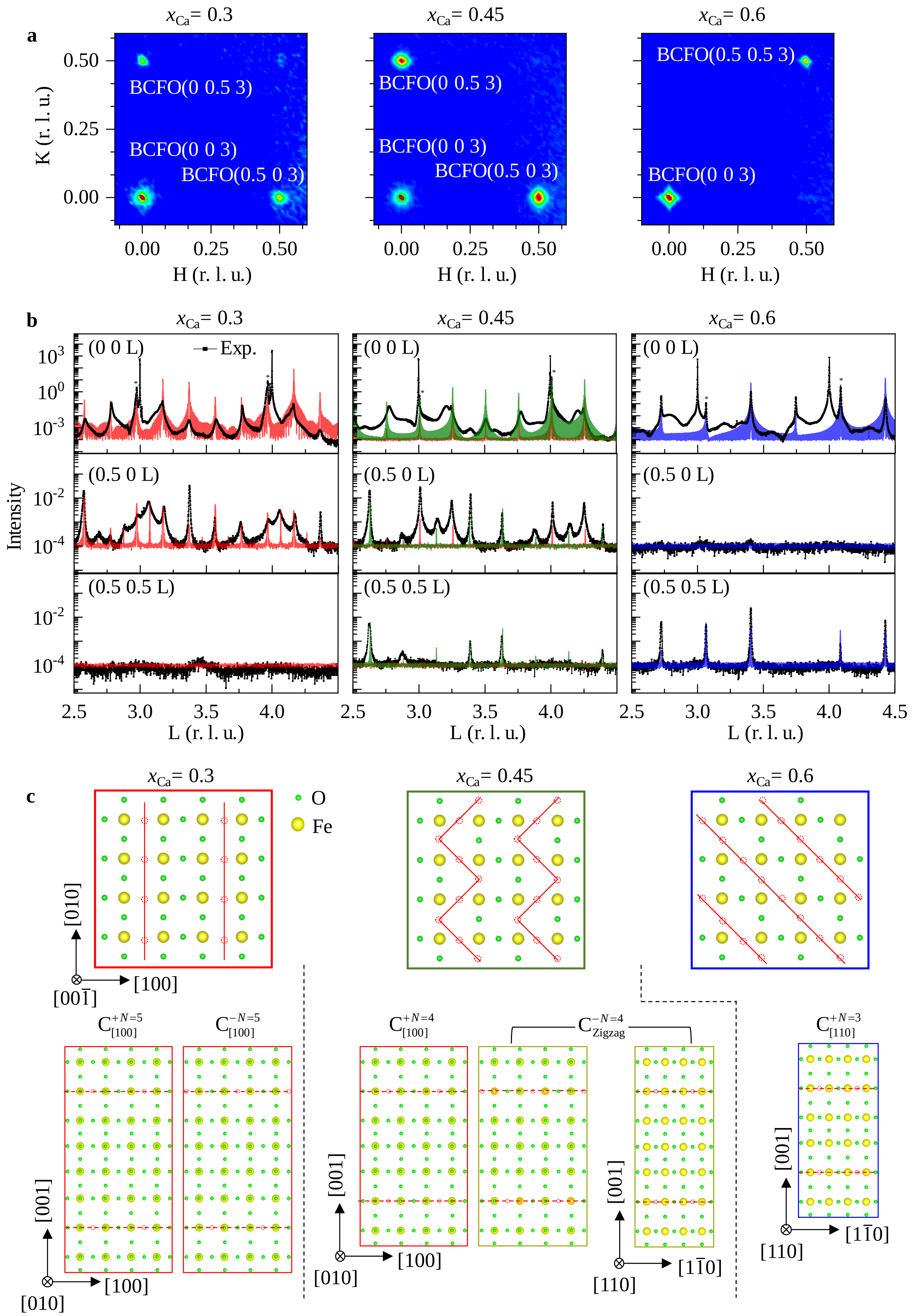

Supplementary Fig. 8: X-ray diffraction of ordered V_O channels and layers in BCFO films. a, HK

reciprocal space maps around BCFO (0 0 3) reflection for x_{Ca} = 0.3, 0.45, and 0.6. **b**, Line scans along the out-of-plane L direction through (0 0 3), (0.5 0 3), and (0.5 0.5 3) BCFO reflections. The asterisks indicate primary diffraction peaks and the reciprocal lattice unit (r. l. u.) is defined as 2π/3.905 $Å^{-1}$. **c**, Model crystal structures with ordered V_O channels used for structure factor calculations. For x_{Ca} = 0.3, <100> V_O channels (red) exist with a two-unit-cell periodicity within the oxygen-deficient layers and a 5-unit-cell periodicity along [001] direction. There are two types of domains regarding V_O channel stacking: i) V_O channels of a deficient layer are stacked in phase at the same in-plane positions of the lower layer ($C_{[100]}^{+\ N=5}$) and ii) V_O channels stacked out of phase with an one-unit-cell in-plane shift ($C_{[100]}^{-\ N=5}$). For x_{Ca} = 0.6, <110> channels (blue) are ordered with a two-unit-cell periodicity within oxygen-deficient layers and a 3-unit-cell periodicity along [001] direction ($C_{[100]}^{+\ N=3}$). V_O channels are stacked on the same in-plane positions. For x_{Ca} = 0.45, a zigzag-type pattern of competing V_O channels emerges (olive). The zigzag-type channels have an in-plane two-unit-cell periodicity, which is consistent with the dimerization observed by STEM along both <100> and <110> zone axes. The out-of-phase stacking of the deficient layers with a 4-unit-cell distance happens along [001] ($C_{\mathrm{Zigzag}}^{-\ N=4}$). In addition to the zigzag-type channels, <100> channel orders seem to mix ($C_{[100]}^{+\ N=4}$). The simulated diffraction patterns based on these models are overlaid in b with the same colors used for the boxes of the corresponding channel models in c. The red dotted circle, red solid lines, and red dotted lines in **c** indicate V_Os, V_O channels, and oxygen deficient layers. The competing domains and stacking faults are scattered within a length scale of the X-ray coherence length less than a micron, since the domain interference effect is necessary to explain the detailed diffraction features. The broad diffusiveness of the diffraction peaks indicates strong disorder in the V_O orders, as seen in the STEM result either. The V_O channels are most likely imperfect and one of four vacancies in V_O channels is expected to be occupied by an oxygen to satisfy the charge balance, as discussed in the theoretical part.

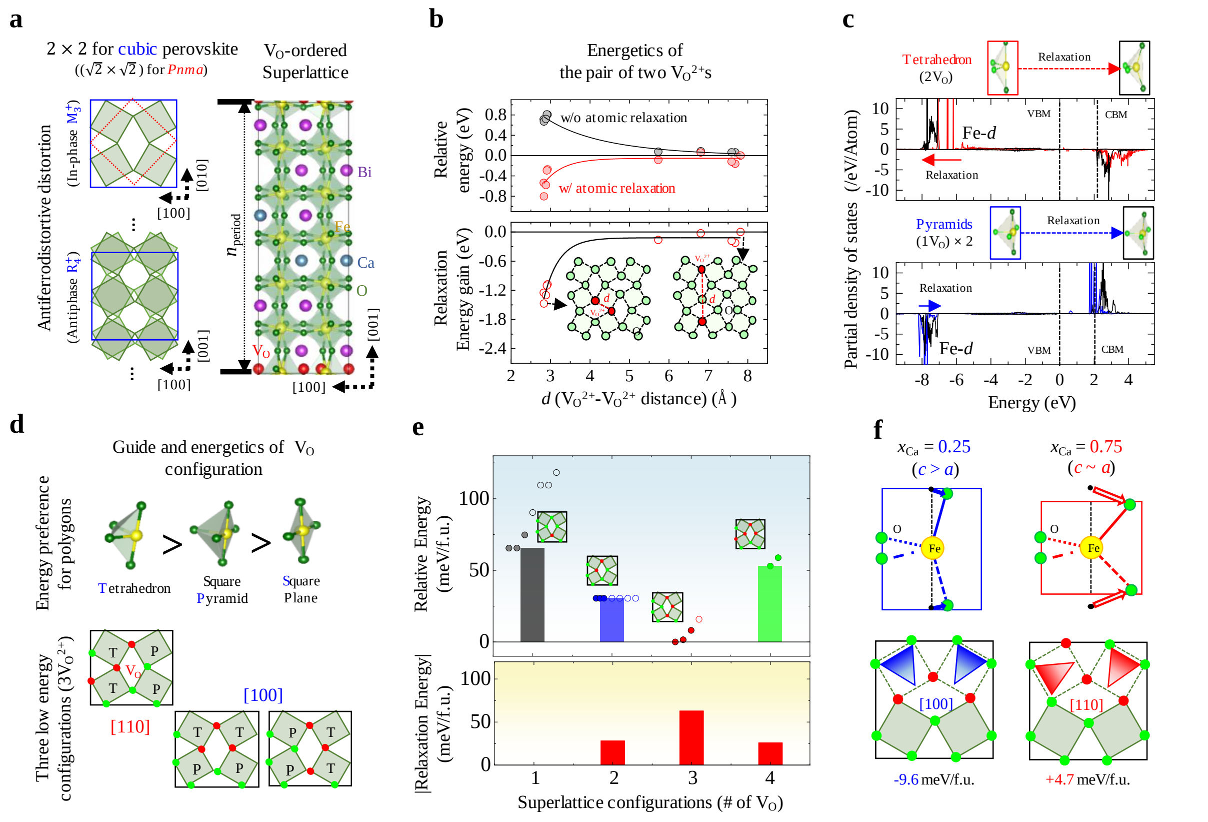

Supplementary Fig. 9: DFT calculation results for the origins of V_O superlattice formation. a, Atomic structures of V_O-ordered superlattice in a representative BCFO (x_{Ca} = 0.25). **b**, Relative total energies and relaxation energies of various V_O^{2+}-V_O^{2+} pairs without or with atomic relaxation (d = V_O^{2+}-V_O^{2+} distance). Energy zero is set to the total energy of the furthest configuration. **c**, Local atomic and Fe-d electronic structures before and after lattice relaxation for (top) the tetrahedron of the clustered case and (bottom) the square pyramids of the far-separated case. **d**, Energy guidelines for the arrangement of a pair of V_Os in terms of polygons. Two square pyramids separated far enough apart were considered for comparison in the same number of ions. Since a square plane has a large energy penalty, it is ruled out. Thus, the three classes of low-energy configurations are allowed for the case of $3V_O$ per 2 × 2 unit cell. They consist only of two tetrahedrons and two square pyramids. Equivalent configurations transformed by the symmetry operations of four-fold rotations and/or translations are classified into the same class. **e**, Energetics and relaxation energies of four oxygen-deficient layers with 1, 2, 3, or 4 V_O per 2 × 2 unit cell for a given x_{Ca} (=0.25). To maintain charge neutrality with Fe^{3+}, the interlayer distance n_{period} decreases with increasing V_O density within the oxygen-deficient layer. (Inset) Schematic diagrams of the energetically lowest structure of in-plane V_O configurations for each superlattice model. The circles representing the relative energies of attempted V_O configurations are filled or open depending on whether all V_Os are only in the in-plane oxygen positions of octahedrons or also in the out-of-plane apical position(s). **f**, Schematic diagrams of relative stability of [100] and [110]

channels depending on symmetric or antisymmetric relaxation at different x_{Ca}. The smaller c-axis lattice constant causes a larger tetrahedral relaxation, which selects the antisymmetric [110] pattern to avoid bumping of two tetrahedrons. The energy value represents the relative energetics, E([100]) - E([110]) at low and high Ca concentrations (x_{Ca} = 0.25 and 0.75). E([100]) and E([110]) are total energies of [100]- and [110]-aligned superlattices, respectively.

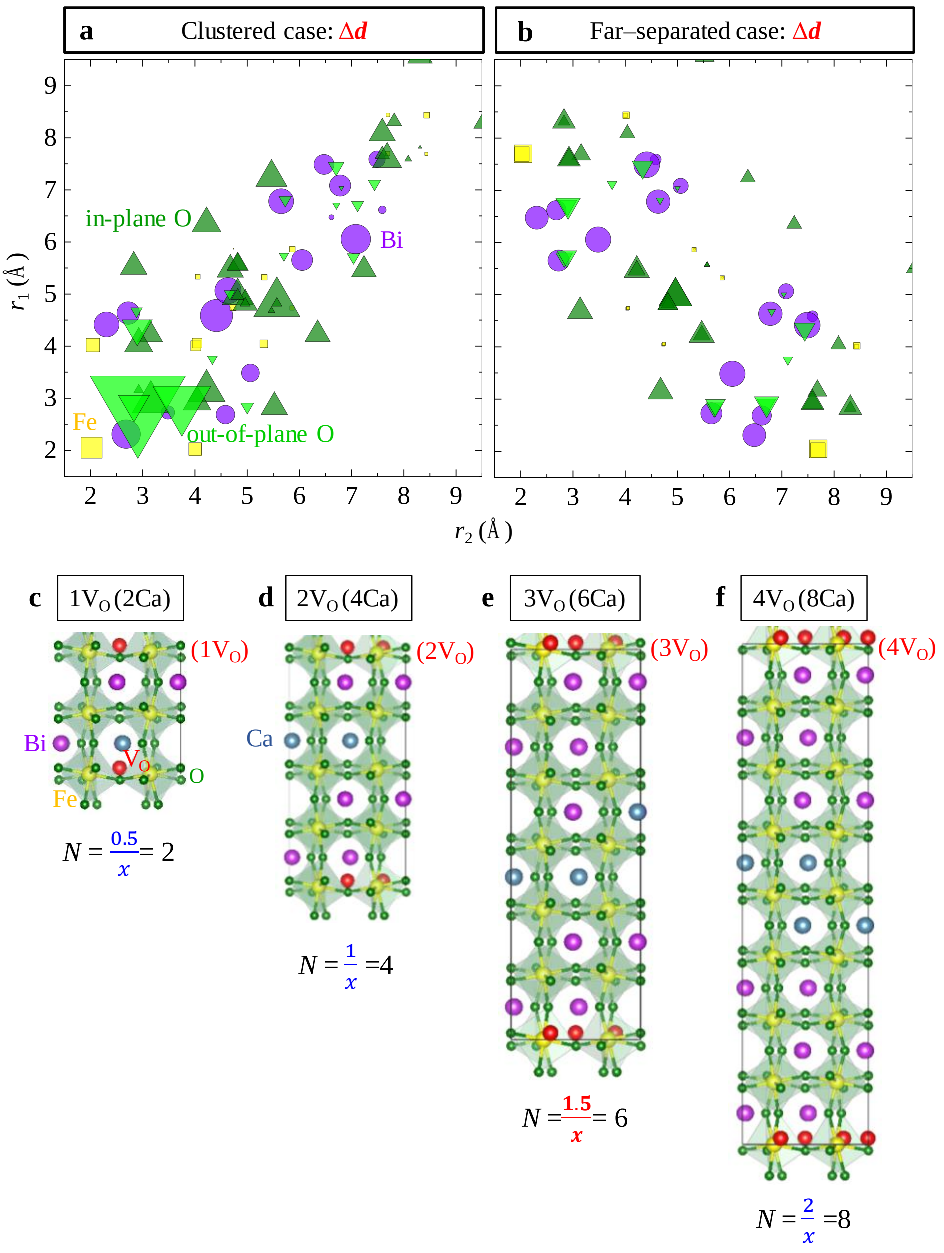

Supplementary Fig. 10: Distribution of the relaxation of all atoms in BCFO and four superlattice models with different periods of oxygen deficient layers. Atomic displacements of all atoms (Ca/Bi, Fe, in-plane O, and out-of-plane O) by two V_O^{2+}s for **a** the clustered case and **b** the far-separated case in BCFO. r_1 (r_2) is the distance to the first (second) V_O^{2+} of all atoms. Here the sizes of the circles, rectangles, and triangles represent the degree of atomic displacement of all atoms. To study the difference in relaxation between the clustered case and the far-separated case, the displacements of all atoms were analyzed and estimated for a representative of each of both the clustered and far-separated cases. The displacement is defined as $\Delta d = |\boldsymbol{r}_{\mathrm{f}} - \boldsymbol{r}_0|$, where (final) $\boldsymbol{r}_{\mathrm{f}}$ and (fixed) $\boldsymbol{r}_0$ are the atomic positions after and before the atomic relaxation, respectively. The atomic displacements in the clustered case are greater than that of the far-separated case, and moreover, the displacements of oxygen ions,

especially out-of-plane oxygens, are found to be the most prominent around the clustered V_Os. This indicates that in the clustered case the relaxation energy gain was achieved mainly by oxygen ions and was large enough to overcome the repulsive Coulomb interaction between V_O^{2+}s. **c-f**, Atomic structures of four period types of V_O layer in a representative BCFO (x_{Ca} = 0.25). **c,** 2-, **d,** 4-, **e,** 6-, and **f,** 8-unit cell periods with the same x_{Ca} and δ are, respectively, constructed for n_{period} = 0.5/x_{Ca}, 1.0/x_{Ca}, 1.5/x_{Ca}, and 2.0/x_{Ca} with 1, 2, 3, and 4 V_Os in the V_O layer.

Supplementary Note 1: A plot of position-time curves on a linear time scale

We provide the same plot of the position-time curves in Fig. 1c on a linear scale for comparison (Supplementary Fig. 11). The plot on a linear time scale shows the low-temperature region clearly, while the plot on a logarithmic time scale displays the high-temperature region more clearly. The experimental data match well with the model fit function except for the initial time regime. The measured phase boundary near the electrode tends to be more slowly moving than the theoretical curve. The nucleation of dark conducting phase takes time after voltage is turned on and this time is now considered as a fitting parameter of t_0 in the model. But the physical origins of determining t_0 are hardly understood yet. There are uncharacterized effects such as the interfacial effect between Pt metal and BCFO film, the extra BCFO region underneath the metal electrode, and the nucleation times for intermediate and dark phase evolution. The fitting was made for a well-defined region of the dark-phase propagation between ~50 μm and 320 μm where the interfacial effect near the electrode is thought to be neglected.

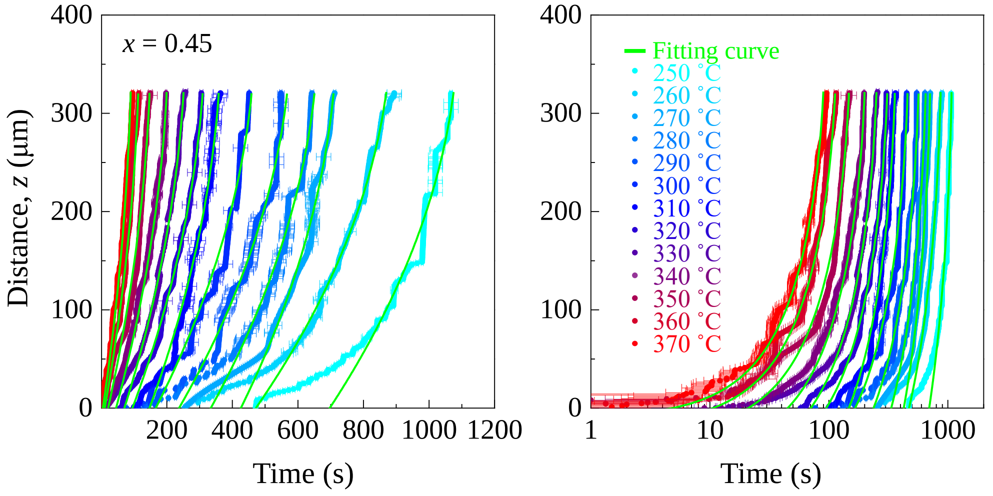

Supplementary Fig. 11: Trajectories of the boundary position (between the intermediate dark-yellow phase and the completely-formed dark phase) as a function of linear (left) and logarithmic (right) elapsed time.

Supplementary Note 2: A more general form of the Nernst-Einstein relation

A more general form of the Nernst-Einstein equation is discussed to describe the diffusion phenomena in non-dilute systems. Before going into details, we need to clarify two different concepts of diffusion coefficients. The chemical diffusion coefficient ($\widetilde{D}$) is a proportionality constant between the gradient in concentration and the diffusion flux described from the Fick's law. On the other hand, the conduction diffusion coefficient (D_σ) is defined from the classical Nernst-Einstein equation ($D_\sigma = \sigma k_B T/nq^2$, with conductivity σ, Boltzmann constant k_B, temperature T, concentration n and charge q). From the Green-Kubo relation, they are defined as [Gomer, R. *Rep. Prog. Phys.* **53**, 917 (1990)]:

$$\widetilde{D} = \lim_{t\to\infty} \frac{1}{2d\langle(\delta N)^2\rangle t}\left\langle\left|\sum_{i=1}^{N} \Delta\boldsymbol{R}_i(t)\right|^2\right\rangle, \tag{1}$$

$$D_\sigma = \lim_{t\to\infty} \frac{1}{2dNt}\left\langle\left|\sum_{i=1}^{N} \Delta\boldsymbol{R}_i(t)\right|^2\right\rangle. \tag{2}$$

N corresponds to the total number of particles, $(\delta N)^2$ is the fluctuation in the particle number, its ensemble average value has a relation of $\langle(\delta N)^2\rangle = \langle N^2\rangle - \langle N\rangle^2$. $\Delta\boldsymbol{R}_i$ is the displacement of ith ion in d-dimensional system after time t, and the time-normalized vector corresponds to the velocity of ith ion. The conventional approaches such as the electrochemical impedance spectroscopy, galvanostatic (or potentiostatic) intermittent titration techniques, and even the electrocoloration method measure the chemical diffusion coefficient $\widetilde{D}$ in terms of the fact that a potential gradient is introduced.

On the other hand, the tracer diffusion coefficient (D^*) is defined as

$$D^* = \lim_{t\to\infty} \frac{1}{2dNt}\sum_{i=1}^{N}\langle|\Delta\boldsymbol{R}_i(t)|^2\rangle. \tag{3}$$

It carries the elementary picture of diffusion for a single particle executing a random walk in d-dimensional space. We notice the correlation terms between different particles are omitted, in contrast to the $\widetilde{D}$ and D_σ. Isotope diffusion experiments using less concentrated ad-particles can give the value of D^*.

The D^* can be related to D_σ by multiplying the correlation factor :

$$H = \lim_{t\to\infty} \frac{\sum_{i=1}^{N}\langle|\Delta\boldsymbol{R}_i(t)|^2\rangle}{\left\langle\left|\sum_{i=1}^{N} \Delta\boldsymbol{R}_i(t)\right|^2\right\rangle}. \tag{4}$$

Provided that a particle is weakly correlated with other particles such as in dilute systems, i.e., $\lim_{t\to\infty}\langle\Delta\boldsymbol{R}_i(t)\cdot\Delta\boldsymbol{R}_j(t)\rangle \to 0$ $(j \neq i)$, D^* becomes equal to D_σ, i.e., $H \to 1$. Observing the deviation of H from 1 by performing an isotope diffusion experiment would be an interesting experiment to test the correlation effect.

Since our experiments are the electrochemical impedance spectroscopy and the electrocoloration approach, we need to focus on the relation between $\widetilde{D}$ and D_σ. As shown in the work by G. E. Murch [G. E. Murch, *Philos. Mag. A* **45**, 685-692 (1982)], the exact and general form of Nernst-Einstein

equation ($\frac{u}{\widetilde{D}} = nq\frac{\partial \ln n}{\partial \mu}$, u is the ionic mobility, n is the concentration of vacancies and μ is the chemical potential of vacancies) looks different from the classical form. But $\widetilde{D}$ can be related to D_σ through a thermodynamic enhancement factor ($W = \frac{\langle N \rangle}{\langle (\delta N)^2 \rangle} = \frac{\partial \mu / k_B T}{\partial \ln n}$), i.e.,

$$\widetilde{D} = W D_\sigma. \tag{5}$$

Under various conditions, the enhancement factor W could be different values [Weppner, W. & Huggins, R. A. Determination of the Kinetic Parameters of Mixed-Conducting Electrodes and Application to the System Li_3Sb. *J. Electrochem. Soc.* **124**, 1569-1578 (1977)]. Of course, it also approaches to 1 in dilute systems. In the as-grown samples without nitrogen annealing, the BCFO samples are mixed ionic-electronic conductors. Also, the electronic conductivity predominates the ionic conductivity because the electronic carriers have a much greater mobility than the ionic species. Since electronic carriers are generated with oxygen vacancies, the electroneutrality condition $z_i \Delta n_i \approx \Delta n_e$ is satisfied in our system (z_i is the charge number of an ionic defect, n_i and n_e are the concentrations of ionic and electronic carriers). In the constraints together with obeying the Raoult's law, the enhancement factor can be written as,

$$W \simeq 1 + {z_i}^2 \frac{n_i}{n_e}. \tag{6}$$

The electrocoloration method quantifies the mobility and thus the diffusivity deduced by the Nernst-Einstein relation corresponds to D_σ. On the other hand, the electrochemical impedance spectroscopy is known as a technique for characterizing $\widetilde{D}$ in the electrolyte regime. By comparing the results of the two methods, we can determine the value of W to be ~2 (See Fig. 1f and Supplementary Fig. 12c). Considering a V_O donates two electrons, n_e is estimated to be a quarter of n_i. Moreover, W is just a weighting factor independent of T in the experimental range and thus the overall tendency for the E_A is not changed.

As abovementioned, the exact value of W can be empirically determined on the assumption that ionic defect density of n_i is appropriately specified, but the estimate is not simple because the spatial distribution of V_Os in BCFO is not fully randomized but they partially play a role of constructing an ordered structure of V_O channels. Taking into account a deviation (δ) of oxygen stoichiometry from the ideally compensated semiconductor, $Bi_{1-x}Ca_xFeO_{3-x/2+\delta}$, we may regard the concentration of only the excess oxygen ions as n_i. Since a single excess oxygen ion is an acceptor for generating two holes ($z_i = 2$) and thus the concentration of produced electronic hole carriers is $n_e = |z_i| n_i$, the value of W becomes 3.

It is worthwhile mentioning that the conductivity of color tracking method could be overestimated by a factor of 3/2. We determined the ionic conductivity $\sigma = nq\mu$ from the measured mobility. When the propagation of the phase boundary between the dark conducting phase (phase III with 0 V_O) and the dark-yellow phase (phase II with 2 V_Os) is examined, the number of moving V_Os are only 2 V_Os per 2×2 pseudocubic unit cells in an oxygen-deficient layer. As compared with the as-grown phase (phase I with 3 V_Os), the change in ionic carrier density is two thirds of n and so does σ. If we accept for the $W \sim 3$, the conductivities from the electrocoloration method and the impedance spectroscopy (Supplementary Fig. 12) are exactly matched each other relying on the correction.

Supplementary Note 3: Nitrogen annealing effect

A previous paper [N. Masó and A. R. West, *Chem. Mater.* **24**, 2127–2132 (2012)] motivates us to anneal our samples in the N_2 gas environment. The paper has reported that the exact stoichiometric $Bi_{0.7}Ca_{0.3}FeO_{2.85}$ ceramics exhibit purely ionic conduction based on impedance spectroscopy. Small amount of off-stoichiometric defects/ions have largely changed conductivity and activation energy barrier. In the Masó and West paper, annealing under oxygen or air environment slightly changed the weight of crystal relative to the nitrogen annealed one, *i.e.*, oxygen annealing: $Bi_{0.7}Ca_{0.3}FeO_{2.85+0.016}$, and air annealing: $Bi_{0.7}Ca_{0.3}FeO_{2.85+0.01}$. Even if the variations look tiny, those give rise to large differences in conductivity and activation energy, indicating an electronic-carrier dominant conduction.

To compare the video method and impedance measurement, we need to make ionic-conduction dominant $Bi_{0.7}Ca_{0.3}FeO_{2.85}$ thin films to determine ionic conductivity by using the standard AC impedance measurement. The as-grown thin film shows an electronic-conduction dominant state without any Warburg-spike feature (red data in Supplementary Fig. 12a). We put a lot of effort into finding the optimal nitrogen-gas annealing conditions for making the ion-conduction dominant state in our $Bi_{0.7}Ca_{0.3}FeO_{2.85}$ films. We successfully obtained the $Bi_{0.7}Ca_{0.3}FeO_{2.85}$ films by annealing at N_2 gas pressure of 10 mTorr and 650 ℃ for 24 hours to suppress electronic carriers induced by off-stoichiometric oxygen atoms. We used platinum interdigitated electrodes to apply an AC bias of 100 mV with a frequency sweep ranging from 1 MHz to 0.1 Hz. We observed a large semicircle and a low-frequency branch (black data in Supplementary Fig. 12a). The large semicircle is attributed from the ionic conduction in bulk, and the branch in the extended low frequency part is a feature of the well-known Warburg spike, arising from the existence of a constant phase element often found in ionic conductors [J. Jamnik and J. Maier, Treatment of the impedance of mixed conductors equivalent circuit model and explicit approximate solutions, *J. Electrochem. Soc.* **146**, 4183–4188 (1999)].

The diameter of the large semicircle represents the ionic resistance of $Bi_{0.7}Ca_{0.3}FeO_{2.85}$. Impedance measurements at different temperatures also show a similar Warburg-diffusion behavior and enable us to estimate the temperature-dependent ionic conductivity (Supplementary Fig. 12b). We evaluated ionic conductivity of $Bi_{0.7}Ca_{0.3}FeO_{2.85}$ thin films using the video analysis by tracking the motion of phase boundary [J. S. Lim *et al.*, Ultrafast collective oxygen-vacancy flow in Ca-doped $BiFeO_3$, *NPG Asia Mater.* **2**, 084412 (2018)]. Comparing the ionic conductivities obtained by two different methods, they match well with each other, revealing the same order of magnitude of conductivity and the same thermal activation energy (Supplementary Fig. 12c). The sample used for the video recording method was not annealed. Nonetheless, the mutual consistency suggests that the visualization technique provides a useful pathway for characterizing ionic conduction properties regardless of the presence of significant electronic conduction.

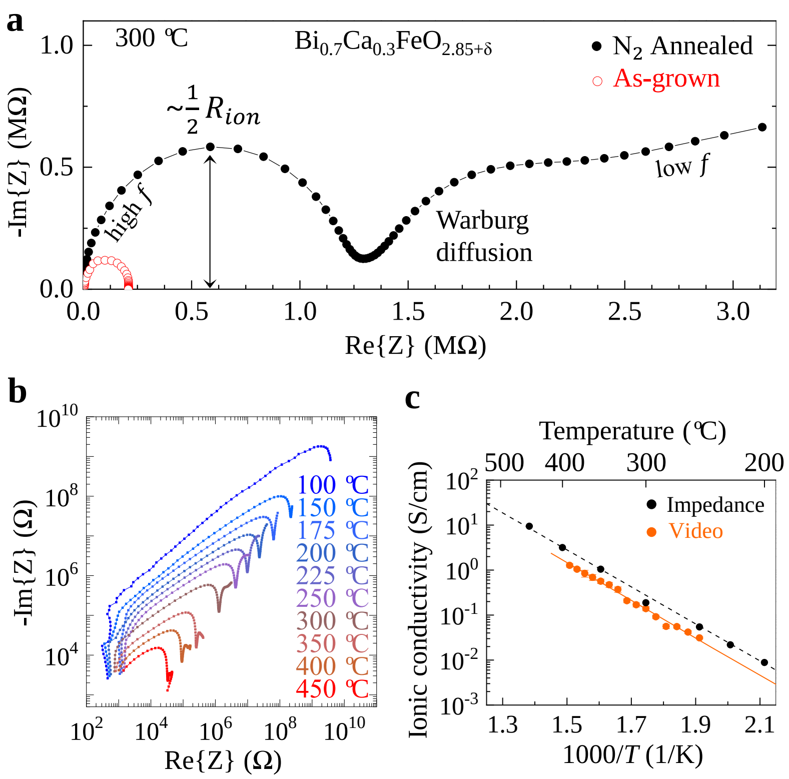

Supplementary Fig. 12: Impedance measurements of $Bi_{0.7}Ca_{0.3}FeO_{2.85+\delta}$. **a**, Nyquist plots of as-grown and N_2 gas annealed $Bi_{0.7}Ca_{0.3}FeO_{2.85+\delta}$ at 300 ℃. **b**, Nyquist plots of N_2 gas annealed $Bi_{0.7}Ca_{0.3}FeO_{2.85}$ with respect to temperature. **c**, A comparison of ionic conductivities obtained from the two different methods, video analysis (orange balls) and impedance measurement (black balls).

Supplementary Note 4: Determination of the oxygen tracer diffusivity

Tracer diffusivity (D^*) of oxygen ions in a BCFO thin film (x_{Ca} = 0.45) was investigated by an oxygen isotope exchange profiling technique using time-of-flight secondary ion mass spectroscopy (TOF-SIMS). A ~50 nm-thick $LaAlO_3$ capping layer was deposited on the ~55 nm-thick BCFO thin film at x_{Ca}=0.45 to determine the tracer diffusivity along the in-plane direction. The sample was scratched with a straight line by a scalpel to open a lateral edge of the thin film to an atmosphere (Supplementary Fig. 4a). Thus, the oxygen exchange was allowed along the in-plane direction [100], while prevented along the out-of-plane direction by the capping layer. The topographic image in Supplementary Fig. 4b shows that the gap was clearly made along the [010]. The width and depth of the gap was ~7 μm and ~200 nm, respectively. The sample was evacuated to <5×10^{-3} Torr and then pre-annealed at 430 °C for 1 hour under a stream of pure oxygen gas (99.995%) of natural isotopic abundance. After the pre-annealing process, the sample was cooled to room temperature and evacuated again to <5×10^{-3} Torr. Then, $^{18}O_2$-rich gas (97.15%) was inserted up to a pressure of ~500 Torr, and annealing at 350°C in the isotope gas environment was conducted for 90 minutes. At last, the sample was cooled down to room temperature. The procedure for the isotope exchange experiment was guided by [Burriel, M. *et al.*, Anisotropic oxygen diffusion properties in epitaxial thin films of $La_2NiO_{4+\delta}$, *J. Mater. Chem.* **18**, 416–422 (2008)].

The ^{18}O concentration profiles were measured using TOF-SIMS 5 (IONTOF GmbH). A beam of 1 keV cesium ions was scanned over an area of 500 × 500 μm^2 to etch the entire $LaAlO_3$ layer and a part of the BCFO thin film. Secondary ion mass spectral images were obtained by scanning over an area of 200 × 200 μm^2 with 256 × 256 pixels using a pulsed beam of bismuth ions accelerated at 30 keV. The mode of negative ion detection was used. Charge compensation was achieved by the use of a flood gun. Supplementary Fig. 4c shows an area mapping of the $^{18}O^-$ intensity near the gap measured by TOF-SIMS. Supplementary Fig. 4d shows line profiles of the $^{18}O^-$ intensity (black) and the sum of $Fe^{16}O^-$ and $Fe^{18}O^-$ intensities (blue) along [100] for the region of black rectangle in Supplementary Fig. 4c. The $^{18}O^-$ profile as a function of position (x) and diffusion time (t) was fitted using a diffusion equation for the one-dimensional semi-infinite medium model developed by [Crank, J. The Mathematics of Diffusion, *Oxford University Press*, (1975)]:

$$C^{'}(x,t)=\frac{\frac{I(^{18}O^-)}{I_{O^-}}-C_{bg}}{C_g-C_{bg}}=\mathrm{erfc}\left(\frac{x}{2\sqrt{D^*t}}\right)-\exp\left(\frac{kx}{D^*}+\frac{k^2t}{D^*}\right)*\mathrm{erfc}\{\left(\frac{x}{2\sqrt{D^*t}}\right)+\left(\frac{k}{D^*}\sqrt{D^*t}\right)\}. \quad (7)$$

$I(^{18}O^-)$ is the $^{18}O^-$ intensity, I_{O-} is the sum of the $^{18}O^-$ and $^{16}O^-$ intensities, C_{bg} is the natural isotopic fraction of ^{18}O, C_g is the isotopic fraction of the gas used for the annealing process, D^* is the bulk oxygen tracer diffusivity, and k is the tracer surface exchange coefficient. C_{bg} and C_g were set to 0.002 and 1. In our analyses, I_{O-} was also set as a fitting parameter. The data points near the scratch (closer than 0.003 cm) were excluded for the fitting to avoid any unwanted edge effects and to focus on pure bulk oxygen diffusion. The deviation of the FeO^- intensities at the edge region also suggests the existence of an edge effect.

As a result, D^* = 2.77(± 0.19)×10^{-9} cm^2/s and k = 1.46(± 0.04)×10^{-9} cm/s were determined at 350 °C in the BCFO film of x_{Ca} = 0.45. The determined diffusivity value is approximately twice lower than the value of conduction diffusivity (D_σ) of 5.9×10^{-9} cm^2/s at the same composition and temperature. A slightly underestimated value seems reasonable as tracer diffusivity does not account for correlation effects due to dilute concentrations of isotope ions.

Depth profiles of $^{18}O^-$ intensity through the entire $LaAlO_3$ capping layer and the upper BCFO part were analyzed (Supplementary Fig. 4e) in three regions with different distances from the gap. The red points represent the average depth profile in the area R1 (shown in the red rectangle in Supplementary Fig. 4c) 30-50 μm distant from the edge of gap. Similarly, the green (blue) points are average depth profiles in the area R2 (R3) 50-70 (70-90) μm distant from the edge of gap. The $^{18}O^-$ intensity abruptly decays within a sputtering time of 200 s. Considering that ~2000 s was taken to fully etch the 50 nm-thick $LaAlO_3$ layer, the sputtering time of 200 s corresponds to ~5 nm. Thus, most of ^{18}O ions injected to $LaAlO_3$ could not diffuse deeper than 5 nm in all the R1, R2, and R3 regions. By etching longer than 2000 s, we can remove the 50-nm-thick capping layer completely and reach the BCFO thin film. In contrast to the almost same profiles of the $LaAlO_3$ capping layer, a gradual decrease in $^{18}O^-$ intensity can be observed from R1 to R2 to R3 regions in the BCFO layer, indicating that the concentration gradient was created by oxygen tracer exchange and diffusion through the in-plane direction.

Supplementary Note 5: Electron diffraction (ED) patterns from TEM measurement

The presence of ordered V_O channels in the BCFO films (x_{Ca} = 0.3, 0.45 and 0.6) was identified by electron diffraction (ED). The (1/2 0 L) diffraction spots (marked with white arrows) in the [010] ED patterns of all BCFO films reveal a two-unit-cell periodicity along the [100] direction (Supplementary Fig. 13a). The (1/2 1/2 L) diffraction spots are also present in the [$\bar{1}$10] ED patterns of the BCFO films with x_{Ca} = 0.45 (faintly visible) and 0.6, indicating a doubling of the periodicity along the [110] direction (Supplementary Fig. 13b).

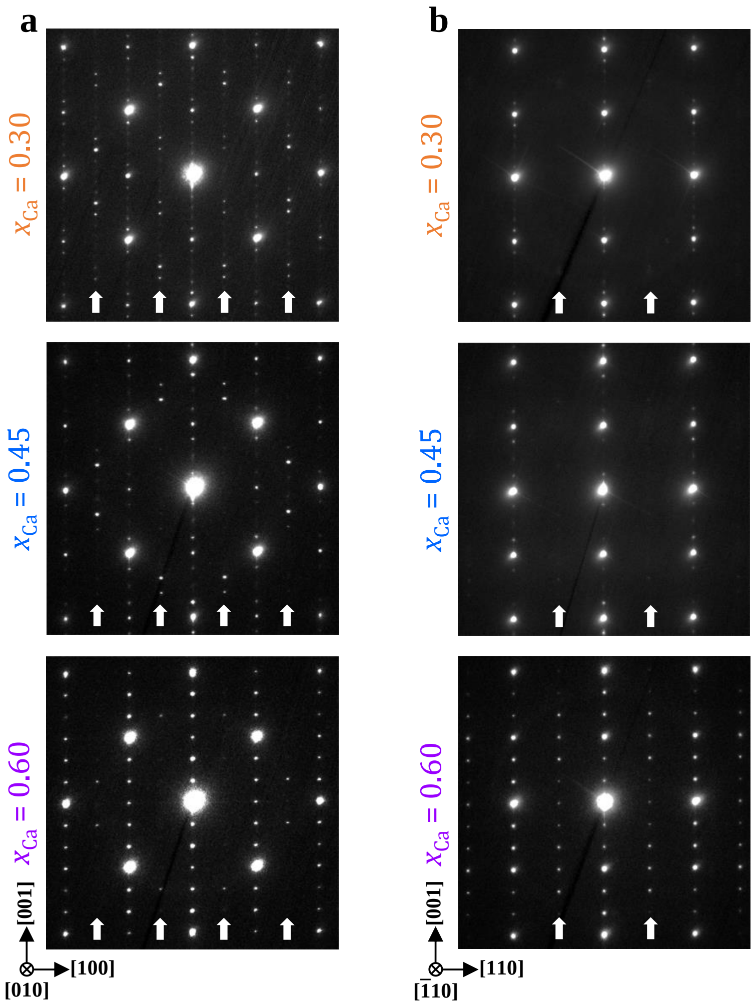

Supplementary Fig. 13: Observation of ordered V_O layers in BCFO films. a, ED patterns along the [010] zone axis. The white arrows in the ED patterns point out the position of the (1/2 0 L) diffraction spots. **b**, ED patterns along the [$\bar{1}$10] zone axis. The white arrows in the ED patterns indicate the positions of the (1/2 1/2 L) diffraction spots. The superlattice reflections along [001] reveal the periodicity of the V_O layers. The periodicity n_{period} is equal to ~6, 4 and 3 for the BCFO films with x_{Ca} = 0.30, 0.45 and 0.60, respectively.

Supplementary Note 6: Theoretical understanding of defect orderings and competition

The atomic structure of as-grown V_O-ordered BCFO superlattice is modeled based on two known conditions: (i) the V_O concentration (δ) should follow $\delta = x_{Ca}/2$ because V_O in BFO is a double-electron donor (V_O^{2+}) compensated by two Ca_{Bi}^{1-} acceptors; (ii) the distance between neighboring oxygen-deficient layers (n_{period}) is experimentally measured to be $n_{period} = 1.5/x_{Ca}$. To meet these conditions, three V_O^{2+}s should be present per 2×2 cubic perovskite unit cell with eight oxygen sites (see Supplementary Fig. 9a for a representative BCFO atomic structure with $x_{Ca} = 0.25$ and $n_{period} = 6$, based on the calculational results below).

To understand the microscopic origins of superlattice formation in BCFO films, we performed first-principles DFT calculations. Regarding the mysteriously ordered V_O layers in BCFO, we identified the following three questions for theoretical investigation: (1) Why does V_O prefer to cluster together and form the ordered layers? (2) Why does the n_{period} follow the rule $n_{period} = 1.5/x_{Ca}$? (3) What are the atomic models for the [100] and [110] patterns of the V_O channels? By answering these questions, we might be able to better understand the experimental findings.

(1) Ordered V_O cluster formation: We first studied various configurations of two V_O^{2+} defects. In our model calculations (see Methods of calculation), the V_O^{2+}-pair configurations at and near the closest distance are found to be more stable than the others. This pairing trend is not common in terms of Coulomb interaction between defects. Thus, we examined the origin of pairing through the atomic and electronic structures. We found that, without atomic relaxation, V_O^{2+} defects prefer to be separated (Supplementary Fig. 9b), in agreement with their repulsive Coulomb interaction. When we allowed atomic position relaxation, however, the $2V_O^{2+}$ cluster was favored over the separated one by 0.86 eV. This means that the atomic relaxation energy in the $2V_O^{2+}$ cluster is large enough to overcome the repulsive Coulomb interaction between V_O^{2+}s (Supplementary Fig. 9b). Our detailed analysis shows that the out-of-plane apical oxygens in the $2V_O^{2+}$ cluster undergo the most pronounced atomic relaxation (Supplementary Fig. 10a and b). This can be understood with crystal field splitting of Fe orbitals in the polyhedra near the $2V_O^{2+}$. While a pair of V_O^{2+} form two five-fold coordinated square pyramids, the $2V_O^{2+}$ cluster naturally relaxes to a four-fold coordinated tetrahedron adjacent to two square pyramids. With the crystal field splitting of the Fe-d orbitals, the relaxation of the tetrahedron requires atomic displacements large enough to reverse the order of t_2 and e bands, whereas the relaxation of the square pyramid only occurs to a small extent with no significant reordering of Fe d orbitals (t_{2g} → d_{xy} and d_{xz}/d_{yz}, and e_g → $d_{3z^2-r^2}$ and $d_{x^2-y^2}$). Our electronic structure analysis demonstrates that the Fe ion centered at the tetrahedron obtains a large energy gain through the atomic relaxation, in contrast to the square pyramid (Supplementary Fig. 9c). Moreover, the square plane is turned out to be highly unstable and thus we consider only three classes of $3V_O$ configurations that are composed of two tetrahedra and two square pyramids, as shown in Supplementary Fig. 9d. These were evaluated to be stable (at least 42-154 meV/f.u.) compared to the other configurations.

(2) $n_{period} = 1.5/x_{Ca}$ *rule*: To evaluate the most favorable value of n_{period} in a representative BCFO at $x_{Ca} = 0.25$ and $\delta = x_{Ca}/2$, we considered 2-, 4-, 6-, and 8-unit-cell periods, corresponding to $n_{period} = 0.5/x_{Ca}$, $1.0/x_{Ca}$, $1.5/x_{Ca}$, and $2.0/x_{Ca}$, respectively (Supplementary Fig. 10c–f). Since the total number of V_Os is conserved, the V_O density within the deficient layer increases accordingly. Supplementary Fig. 9e shows that the 6-unit-cell period corresponding to $n_{period} = 1.5/x_{Ca}$, which removes 3 out of the 8 oxygens in the oxygen-deficient layer, is the most stable one as a result of the optimization of atomic relaxation energy gain (bottom of Supplementary Fig. 9e).

(3) *[100] vs [110]*: Using our atomic model of the $n_{period} = 1.5/x_{Ca}$, we compared the energy stability of

the [100] and [110] V_O channels for low x_{Ca} (= 0.25) and for high x_{Ca} (= 0.75). Even when we varied the entire contents of V_O in the sample by control of x_{Ca}, the density of V_O remained equal within each 2D oxygen-deficient layer. Supplementary Fig. 9f shows that the [100]-aligned V_O^{2+} channel is favored at x_{Ca} = 0.25, while the [110]-aligned V_O^{2+} channel is favored at x_{Ca} = 0.75. This agrees well with the experimental results. Interestingly, the relative stability of [100] and [110] channels varies due to the energy penalty for the tetrahedral relaxation patterns depending on x_{Ca}. At high Ca doping, the reduction of the *c*-axis lattice constant results in a large tetrahedral relaxation, causing the two tetrahedrons to bump into each other in the symmetric [100] configuration. Therefore, the V_O^{2+} channel pattern of BCFO is easily understood to be determined by symmetric or antisymmetric tetrahedral relaxation depending on x_{Ca}.

Supplementary Fig. 14 shows DFT calculation results for energy comparison between the [110] and [100] channel ordering configurations. We compared the two energies at two selected values of x_{Ca} = 0.25 and 0.75 for three in-plane lattice constants (2*a*). It can be seen that the higher Ca doping ratio is more likely to favor the [110] channel order. All the BCFO films are grown in $SrTiO_3$ substrates, so the in-plane lattice parameters are fixed to 2×3.905 = 7.81 Å. At this value of in-plane lattice parameter, the BCFO at x_{Ca}=0.25 prefers to stabilize the [100]-channel order while the BCFO at x_{Ca}=0.75 prefers the [110]. The theoretical evaluation of energy competition shows an excellent agreement with the experimental results.

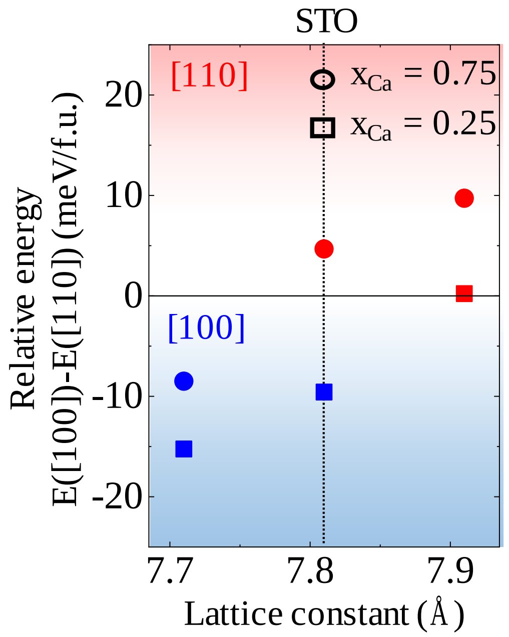

Supplementary Fig. 14: Energy comparison of the two competing oxygen-vacancy channel ordering structures. The energy value represents the relative energetics, *E*([100]) - *E*([110]) at low and high Ca concentrations (x_{Ca} = 0.25 and 0.75). *E*([100]) and *E*([110]) are total energies of [100]- and [110]-aligned superlattices, respectively.

Supplementary Note 7: Structural model parameters and domain population

In structure factor calculations, we first employed the pseudocubic perovskite structure without octahedral tilts and Fe ion shifts for simplicity. And, we assumed the random distribution of Ca ions at Bi sites so that average values of atomic form factor were used for the scattering from A-site as $x_{ca}f_{ca}+(1-x_{ca})f_{Bi}$, where $f_{ca(Bi)}$ indicates atomic form factor for Ca (Bi). The atomic form factors were obtained from a reference [Brown, P. J., Fox, A. G., Maslen, E. N., O'Keefe, M. A., & Willis, B. T. M. Intensity of diffracted intensities, Int. Tables Crystallogr. C, 554-595 (2006).]. And then, we introduced our models of V_O channels as illustrated in the Supplementary Fig. 8.

According to HK maps in Supplementary Fig. 8a, all films are fully strained and the in-plane lattice parameters are identical to those of substrate. The average *c*-axis lattice parameter (c_{avg}) was obtained from the lattice parameter of the supercell formed by the V_O ordering. We also considered local *c*-axis lattice expansion at oxygen-deficient layers, while maintaining the c_{avg} value (smaller than the expanded *c*) for the other layers. The *c*-axis lattice parameters were decided based on local tetragonal *c*/*a* distortion values (Supplementary Fig. 5) and by comparing with the experimental X-ray diffraction data (Supplementary Fig. 8). In the L scans at half-ordered in-plane positions, additional peaks appear in the middle of BCFO film peaks in (0 0 L) scans, which could be explained by domains with V_O channels stacked in an out-of-phase fashion with an in-plane one-unit-cell shift. The relative populations of V_O domains were obtained by the comparison between the structure factor calculation with spatial coherence of possible domain distributions and the intensity modulation in L scans. The resultant parameters and possible domains are summarized in the Supplementary Table 1. A spatial coherence between multiple V_O domains arising from different stacking orders of the oxygen deficient layers is essential for explaining the intensity modulation in the L scans (Supplementary Fig. 15).

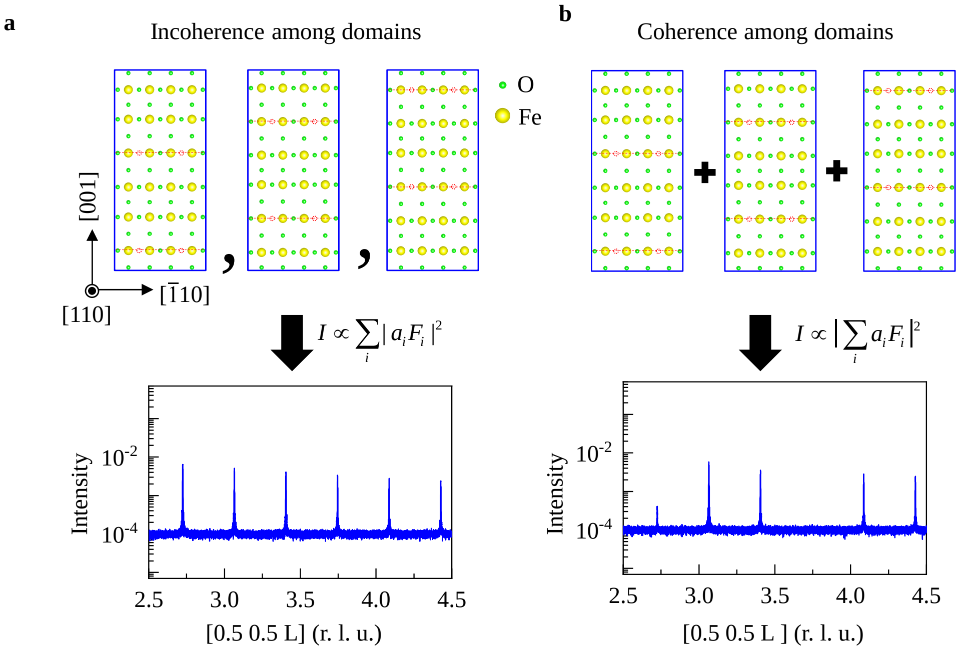

Supplementary Fig. 15: Spatial coherence between domains with different stacking order of the oxygen deficient layers. a, Monotonic change appears in the case of spatial incoherence. **b**, Relative peak intensity become altered in the case of spatial coherence. Here, *I*, a_i and F_i stand for intensity of diffraction, population of i_{th} domain, and structure factor of i_{th} domain. The red dotted circle and red dotted lines indicate V_O and oxygen deficient layers.

x_{Ca}	0.3			
Thickness (nm)	a (= b) (Å)	c_{avg} (Å) (c_{avg}/a)	c_{V_O} (Å) (c_{V_O}/a)	c_o (Å) (c_o/a)
108	3.905	3.938 (1.008)	4.395 (1.125)	3.824 (0.979)

Relative domain population (%)

$C^{+N=5}_{[100]}$ | $C^{-N=5}_{[100]}$

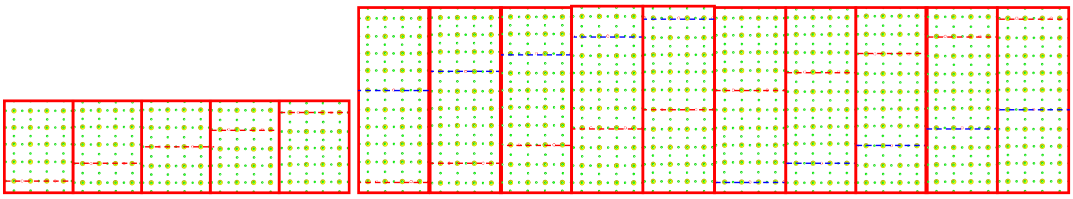

0 | 10.7 | 0 | 0 | 13.5 | 0 | 0 | 12.6 | 12.6 | 25.3 | 12.6 | 12.6 | 0 | 0 | 0

x_{Ca}	0.45			
Thickness (nm)	a (= b) (Å)	c_{avg} (Å) (c_{avg}/a)	c_{V_O} (Å) (c_{V_O}/a)	c_o (Å) (c_o/a)
125	3.905	3.894 (0.997)	4.186 (1.072)	3.797 (0.972)

Relative domain population (%)

$C^{+N=4}_{[100]}$ | $C^{-N=4}_{Zigzag}$

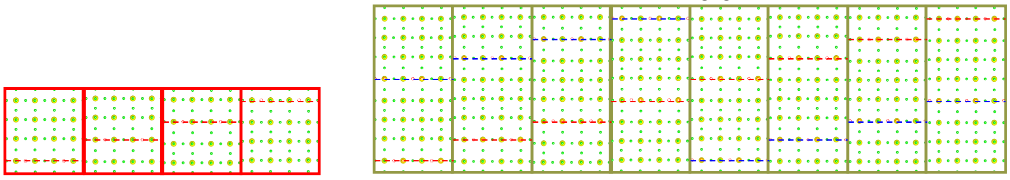

0 | 11.8 | 23.6 | 23.6 | 17.6 | 0 | 11.7 | 0 | 11.7 | 5.9 | 0 | 0

x_{Ca}	0.60			
Thickness (nm)	a (= b) (Å)	c_{avg} (Å) (c_{avg}/a)	c_{V_O} (Å) (c_{V_O}/a)	c_o (Å) (c_o/a)
103	3.905	3.822 (0.979)	4.128 (1.057)	3.669 (0.940)

Relative domain population (%)

$C^{+N=3}_{[110]}$

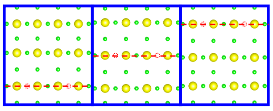

50 | 50 | 0

Supplementary Table 1: Parameter values for structure factor calculations. The parameter values and the relative domain population used in structure factor calculations. The c_{V_O}, c_o and c_{avr} correspond to c-axis lattice parameters of oxygen-deficient layers, the layers without V_O channels and their average. Each relative population of domains corresponds to the inset unit-cell schematics above each value. The red dotted lines indicate oxygen-deficient layers and the blue dotted lines indicate the ones with the in-plane one-unit-cell shift.

Supplementary Note 8: Analysis of the propagation of intermediate phase region

We have observed three color phases in the color diagram: pale yellow (as-grown), dark yellow (intermediate) and dark black (completely formed) phases. We have mainly dealt with the distinct color boundary between dark-black and dark-yellow phases to characterize the motion of oxygen vacancies in the main manuscript. Here, we analyze the front propagation of the intermediate phase as well.

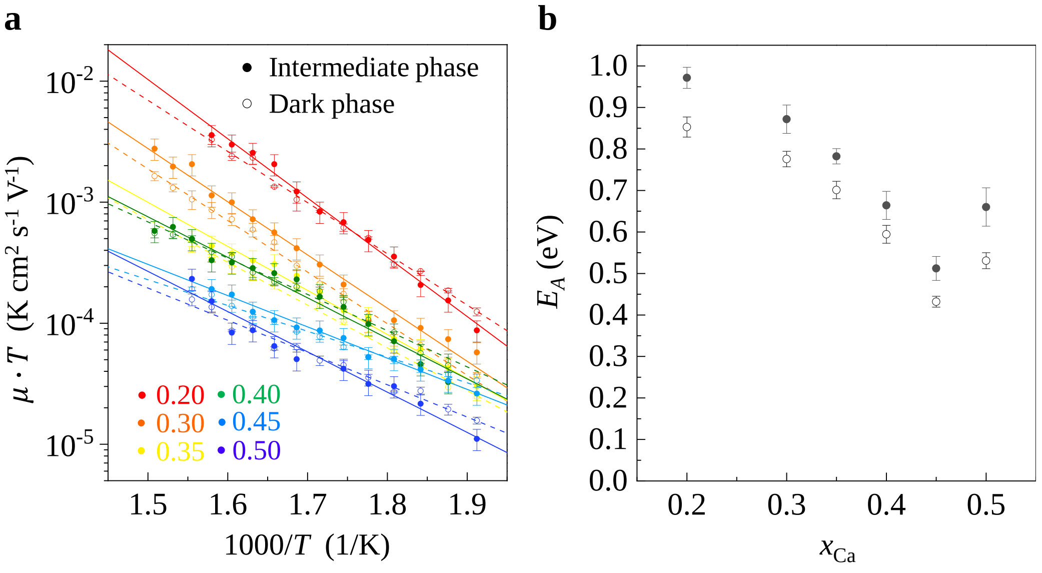

Supplementary Fig. 16: Temperature dependence of the phase front mobility for the intermediate and dark phase propagations. a, Arrhenius plots of the product of collective V_O mobility (μ) and temperature (T) versus reciprocal temperature for different x_{Ca} = 0.2 ~ 0.5. **b**, The activation barrier E_A with respect to x_{Ca}, which is obtained by the slopes of linear fit lines in a. Solid and open symbols (solid and dashed lines) correspond to the intermediate phase and the dark conducting phase, respectively. We note the boundary between the intermediate phase and the pristine phase is not clearly seen in BCFO films at x_{Ca} = 0.1 or 0.6.

The color boundaries between the intermediate dark-yellow phase and the original yellow phase at different temperatures are highlighted by green dashed lines in the Supplementary Fig. 2. Each of the lines was obtained by fitting the position-time (x_{int} vs. t_{int}) curve of the boundary of intermediate phase to the same equation which was used for the analysis of dark-phase propagation. We can estimate the ionic mobility μ from the experimentally determined $\Delta t_{int}=t_{int}-t_0$ through the relation $\mu = L^2/(2\Delta t_{int}V)$. L stands for the channel length of 400 μm and V is the applied voltage. The error bar of Δt_{int} was set to be 20 % of the corresponding value. The ionic mobility of intermediate phase propagation shows a similar value compared to that of the dark-phase propagation at the examined x_{Ca}s (Supplementary Fig. 16a). The product of temperature and mobility is also linearly proportional to inverse of temperature, indicating E_A from the slope. The E_A of ionic transport for the intermediate phase is roughly ~ 0.1 eV higher than that of the dark phase (Supplementary Fig. 16b). The value of E_A can be larger as much as ~0.4 eV in the case of individual hopping, according to the density functional calculation result. Accordingly, it suggests the correlation effect cannot be fully neglected in the intermediate phase containing relatively sparse oxygen ions within the V_O channels.
