# Supplementary material for: Critical ionic transport across an oxygen-vacancy ordering transition
Source: Nat Commun. 2022 Sep 1;13:5130. doi: 10.1038/s41467-022-32826-8 (PMC9437025; doi:10.1038/s41467-022-32826-8)
Supplement: Supplementary file 3 — Lasing Reporting Summary [file 41467_2022_32826_MOESM3_ESM.pdf]

nature research

Corresponding author(s): CHAN-HO YANG, YONG-HYUN KIM, MARTA D. ROSSELL

Lasing Reporting Summary

Nature Research wishes to improve the reproducibility of the work that we publish. This form is intended for publication with all accepted papers reporting claims of lasing and provides structure for consistency and transparency in reporting. Some list items might not apply to an individual manuscript, but all fields must be completed for clarity.

For further information on Nature Research policies, including our data availability policy, see Authors & Referees.

ü Experimental design

Please check: are the following details reported in the manuscript?

1. Threshold

Plots of device output power versus pump power over a wide range of values indicating a clear threshold

- [ ] Yes
- [x] No

Our manuscript doesn't include any lasing result.

2. Linewidth narrowing

Plots of spectral power density for the emission at pump powers below, around, and above the lasing threshold, indicating a clear linewidth narrowing at threshold

- [ ] Yes
- [x] No

Our manuscript doesn't include any lasing result.

Resolution of the spectrometer used to make spectral measurements

- [ ] Yes
- [x] No

Our manuscript doesn't include any lasing result.

3. Coherent emission

Measurements of the coherence and/or polarization of the emission

- [ ] Yes
- [x] No

Our manuscript doesn't include any lasing result.

4. Beam spatial profile

Image and/or measurement of the spatial shape and profile of the emission, showing a well-defined beam above threshold

- [ ] Yes
- [x] No

Our manuscript doesn't include any lasing result.

5. Operating conditions

Description of the laser and pumping conditions
Continuous-wave, pulsed, temperature of operation

- [ ] Yes
- [x] No

Our manuscript doesn't include any lasing result.

Threshold values provided as density values (e.g. W cm^{-2} or J cm^{-2}) taking into account the area of the device

- [ ] Yes
- [x] No

Our manuscript doesn't include any lasing result.

6. Alternative explanations

Reasoning as to why alternative explanations have been ruled out as responsible for the emission characteristics
e.g. amplified spontaneous, directional scattering; modification of fluorescence spectrum by the cavity

- [ ] Yes
- [x] No

Our manuscript doesn't include any lasing result.

7. Theoretical analysis

Theoretical analysis that ensures that the experimental values measured are realistic and reasonable
e.g. laser threshold, linewidth, cavity gain-loss, efficiency

- [ ] Yes
- [x] No

Our manuscript doesn't include any lasing result.

8. Statistics

Number of devices fabricated and tested

- [ ] Yes
- [x] No

Our manuscript doesn't include any lasing result.

Statistical analysis of the device performance and lifetime (time to failure)

- [ ] Yes
- [x] No

Our manuscript doesn't include any lasing result.
